# Online cultural experiences for mental health in people aged 16–24: a qualitative analysis of multisource data from a randomised controlled trial

**DOI:** 10.1136/bmjopen-2025-105217

**Published:** 2026-04-28

**Authors:** Rebecca Syed Sheriff, Evgenia Riga, Bessie O’Dell, Louise Chandler, Helen Adams, Margaret Glogowska

**Affiliations:** 1 University of Birmingham—Edgbaston Campus, Birmingham, UK; 2 Department of Psychiatry, University of Oxford, Oxford, UK; 3 Gardens, Libraries and Museums, University of Oxford, Oxford, UK; 4 Nuffield Department of Primary Care Health Sciences, University of Oxford, Oxford, UK

**Keywords:** Adolescent, MENTAL HEALTH, Depression & mood disorders, Anxiety disorders

## Abstract

**Abstract:**

**Objective:**

To further understand young people’s perceptions of using online arts and culture and how it impacts on mental health.

**Methods:**

This qualitative study was embedded in a proof-of-principle randomised controlled trial (RCT) comparing the effectiveness of two different online arts and culture experiences on mental health in young people (aged 16–24 years). The RCT compared the Ashmolean website (Ash) a generic museum website and Ways of Being (WoB), a codesigned stories based web experience. Three sources of data were analysed; focus group transcripts, free text responses and viewpoints. We adopted an interpretive phenomenological approach allowing deductive and inductive hybrid thematic analysis to gain critical insight into how young people make sense of phenomena relating to mental health in a complex context within a critical realist paradigm.

**Results:**

In total, 117 free text responses relevant to the interventions were received. The first focus group was attended by seven Ash participants and the second by six WoB participants. A total of 108 separate viewpoints were entered. The main themes identified across sources were of human connection, the content and journey of the online experience, the features, setting and when it was used, positive mental health impacts and neutral/negative effects. Positive mental health impacts were often described in association with human connection in WoB participants. Neutral and negative effects were more commonly described in participants allocated to Ash.

**Conclusion:**

Continued development of online arts and culture for diverse populations using participatory and mixed methods to identify potential mechanisms are promising future areas of mental health research.

**Trial registration number:**

NCT04663594; Results

**STRENGTHS AND LIMITATIONS OF THIS STUDY**

- Multisource data captured at different timepoints for participants allocated to two different interventions as part of a randomised controlled trial, allowed for further illumination, as to which aspects of online arts and culture impact on mental health in young people of diverse backgrounds.
- Use of mixed methodologies sheds further light on the potential mechanisms by which online arts and culture impact on mental health as identified by young people of diverse backgrounds.
- The unique context of COVID may have led to mental health and life challenges that may have reduced applicability to young people in different contexts.

## Introduction

 The mental health of young people (YP) is a major public health concern,^1^ given its impact on all aspects of life, including relationships, education, work (productivity and presenteeism) long-term health outcomes and even longevity.^2 3^ Three quarters of all mental disorders emerge before the age of 24^4 5^ and evidence suggests high levels of unmet need.^6^ In addition to the impact on YP’s lives, the cost for the public mental health services and associated costs of sickness absences across the lifespan is increasing.^7^ Given the multifaceted issue of poor mental health, poor access to mental health services, social stigma (including fear of stigma) and inadequate care, that YP—particularly those from under-represented backgrounds—face,^8 9^ the need emerges for alternative sources of mental health support. A promising source of support is arts and culture, which has been found to be beneficial for health, mental health and longevity.^10^ Mechanisms such as emotional activation, interaction, imagination and social and cognitive stimulation are suggested as potential mechanisms through which engagement with the arts could support mental health.^11 12^

Evidence suggests that culture and the arts impact positively on mental and physical health and well-being.^13^,^18^ The mechanisms by which this might occur include emotional activation, aesthetic engagement, interaction, social and cognitive stimulation, sensory activation and imagination.^13 18^ However, engagement with culture and the arts appears to be poorest for those who might benefit the most.^19^

A recent service evaluation of an arts-based social prescribing intervention for adolescents aged 13–16 years showed a significant improvement in participants’ well-being and resilience following the intervention.^20^ Face-to-face interventions, however, come with barriers, for example, geographical location and access. In contrast, digital interventions are more accessible, making them an inclusive and cost-effective option for supporting the mental well-being of YP from diverse backgrounds.^21 22^

Despite the benefits, a systematic review of community interventions for mental health revealed that most research in this field focuses on art therapy in older populations.^23^ Research on online arts and culture focusing on a target population of YP is scarce. A systematic review further demonstrated that a study design involving a collaborative development process with YPs was both empowering for the codesigners and has greater likelihood to reach the target population more widely.^24^

A proof-of-principle randomised controlled trial (RCT)^25^ was conducted to compare the impact of two different online cultural resources on mental health, specifically positive and negative affect and psychological distress. This compared a typical museum website (the Ashmolean museum website) and Ways of Being (WoB), a codesigned online cultural experience. This qualitative study was embedded in the RCT. The study aim was to gain insight into YP’s experiences of using the online cultural interventions and their perceived impact on mental health and well-being. Specifically, the objectives of this study were to gain further understanding of the subjective experience of using online arts and culture, the aspects of online arts and culture experiences perceived to impact on mental health and well-being and how these impacts are perceived to occur in a diverse sample of YP.

## Methods

A multisource qualitative study embedded within a proof-of-principle RCT^26^ was chosen as the methodology to gain further understanding of the subjective experience of using online arts and culture for diverse YP, the perceived impact on mental health and well-being and the mechanisms perceived to lead to these impacts. The trial received approval by the University of Oxford Central University Research Committee; approval reference number R70187/RE007. All participants were given £10 for every hour of participation. Data security followed institutional guidance. Full details on the trial methods and results are found in the relevant publications.^27 28^

### Public and patient involvement

Public and patient involvement (PPI) in these studies commenced prior to the outset and informed the preferred term for the target population as young people, ‘YP’, as well as this study design, topic guide and interpretation of the results. PPI members were involved in stakeholder meetings as well as exploratory discussions. PPI members thought that people from the target population should be an active part of the strategy to understand the use of these resources for mental health benefit.

### Context

This multisource qualitative study was embedded in an online proof-of-principle RCT.^27^ The trial design was parallel group with an allocation ratio of 1:1. The participants were allocated to either a codesigned online cultural experience (intervention), called WoB or a typical museum website (comparator)—the Ashmolean Museum (Ash). The trial protocol^28^ and trial itself^27^ are described in more detail elsewhere. The trial included a series of outcome measures and questionnaires that were completed at baseline, during a 3-day intervention phase and at a 6-week follow-up point (exit).

### Sampling

Trial participants were aged 16–24 and based in the UK (or 18–24 if based overseas). In the UK, YP aged 16 and 17 years are able to give informed consent without parental involvement as ‘competent youths’. Trial participants were recruited online via social media, school and university student organisations. Those who expressed an interest received a participant information sheet and informed consent form and were asked to complete the latter electronically prior to their enrolment. Recruitment occurred between late 2020 and early 2021.

Trial participants who gave consent to be approached were invited to take part in focus groups. Consent was provided electronically. The invitees were purposively selected based on key demographic factors such as age, gender, ethnicity and key socioeconomic factors such as education and occupation. Focus group consent was provided and voice-recorded prior to the focus group discussion taking place.

### Procedure

#### Intervention

Participants were requested to engage with WoB or Ash (depending on allocation) at least once a day for at least 3 consecutive days from the day following allocation, for around 30 min a day. In WoB, stories were represented by a title on the opening page, for example, ‘Being Ibrahim’. Once entered, these led into a long-form story (which could be listened to or read) interspersed with visual media and then led further into ‘deeper’ stories, which were interconnected. Alongside the stories was a comments tool that enabled participants to add their own viewpoint to a variety of viewpoint prepopulated during coproduction, enabling participants to express their responses anonymously. The features of WoB are illustrated in a video walk-through (www.youtube.com/watch?v=mQLFL4Tm-v8). A Standards for Reporting Qualitative Research (SRQR) reporting checklist has been completed (see online supplemental material 2).

Ash is a typical museum website owned by the Ashmolean Museum of Art and Archaeology at the University of Oxford. Participants who were allocated to Ash were directed towards the Ashmolean from Home webpage (https://ashmolean.org/ashmoleanfromhome). This section of the Ashmolean Website was specifically created as an online substitute for the museum visit and material curated by museum specialists, with a focus on art movements or styles. Unlike WoB, the emphasis was on the objects themselves rather than human stories.

#### Data collection

Quantitative data were collected at baseline, daily on 3 consecutive days, at day 5 and week 6. The qualitative data described in this study comprise three sources of data, viewpoints, free text responses and focus groups.

Viewpoints were a feature of WoB that allowed participants to write text anonymously reflecting on content, which was then made available alongside other viewpoints for other participants to read.

Free-text responses were collected at the 6-week follow-up exit assessment of the trial. All participants had free-text options to leave feedback on their allocated online cultural experience at the 6-week timepoint (exit). There were two sets of free-text response questions. The first set was relevant to the intervention. Participants were invited to share any noticeable positive or negative impacts of their allocated cultural experience and their suggestions on how their allocated intervention could be improved for mental health. The second set of questions sought participant views on their research experience and any suggested improvements. Feedback on the research itself will not be reported here.

Two ‘focus groups’ were conducted shortly after completion of the study—one for each intervention group. These two online focus groups took place on Zoom. They were both audio-recorded and transcribed. The topic guide (online supplemental material 1) was codesigned with under-represented youth PPI members. One focus group was attended by seven Ash participants and the second by six WoB participants. Focus groups were audio-recorded and transcribed for analysis.

### Analysis

Data were stored according to local data security procedures and deidentified prior to sharing. They were analysed using a reflexive thematic analysis within a critical realist paradigm. This approach is suited to exploring nuanced, subjective experiences.^29^ Five members of the research team carried out the analysis, RJSS (psychiatrist and research lead), ER (research assistant), BO (DPhil student), HA (museum worker) and MG (senior qualitative researcher). First, a preliminary analysis of the data sources was conducted separately, viewpoints by HA, free text responses by ER and focus group transcripts by BO. ER and RJSS then familiarised themselves with all of the data again by reading, cleaning and organising the datasets separately. RJSS and ER generated initial themes and subthemes by clustering participant quotes with similar meaning across the free-text responses and the focus group transcripts. Data were coded and organised into themes using NViVo.^30^ Inductive thematic analysis allowed for free exploration of the data sources, leading to the identification of themes and subthemes.^29^ Initial themes were formed based on focus group transcripts and free text. Viewpoints were analysed and used to add depth to the initial themes and subthemes as the viewpoints only applied to WoB participants. Deductive thematic analysis was utilised to explore possible links to the quantitative findings of the RCT,^27^ in particular, negative affect scores across the intervention phase and at 6 weeks as well as previous work on potential mechanisms.^31^ This hybrid of inductive and deductive thematic analysis is a highly systematic and robust process, leading to the production of rigorous results^32^ complementary to mixed-methods studies.^33^

## Results

### Randomised controlled trial

A total of 463 participants were randomised, 232 to Ash and 231 to WoB. Quantitative results are described in more detail elsewhere,^27^ but in brief over the intervention phase (an aggregate score including all postallocation timepoints to day 5), a group difference was apparent in favour of WoB for negative affect (WoB-Ash n=448, NA −0.158, p=0.010), but no differences were detected for positive affect or psychological distress and differences were not detected at week 6. Group differences in negative affect in favour of WoB were detected in specific subgroups; ethnic minorities, males, 18–24-year olds and regular users of online arts and culture at baseline. Across participants (from both groups), mean psychological and negative affect improved between baseline and 6 weeks despite increased COVID-19 restrictions. Trial recruitment was rapid, retention high and feedback positive with broad geographical, occupational and ethnic diversity.

### Qualitative analysis

#### Participants and units of study

In total, 349 participants completed the exit survey. 172 had been allocated to Ash and 177 to WoB (demographics in table 1). In all, 324 free-text responses were received; 117 of which were relevant to the interventions. Out of these, 61 were shared by WoB participants and 56 were from Ash participants.

**Table 1:** Characteristics of those who completed the exit survey

	Total	Ash	WoB
Overall	349	172	177
Age			
16–17	33	11	22
18–24	316	161	155
Gender			
Female	169	111	58
Male	173	105	68
Other/prefer not to say	7	3	4
Ethnicity			
Asian British (Indian Pakistani or Bangladeshi)	28	16	12
Black/Black British (Caribbean African or Other)	21	10	11
Chinese/Chinese British	5	4	1
Mixed race (Other)	15	6	9
Mixed race (White and Black/Black British)	12	8	4
Other/prefer not to say	6	3	3
White (British Irish or Other)	262	125	137
Occupation			
At school	41	20	21
In full-time employment (not studying)	84	46	38
In part-time employment (not studying)	70	35	35
On furlough (not studying)	12	3	9
Other	12	6	6
Studying and not working	74	38	36
Studying and working	46	21	25
Unable	1	0	1
Relationship			
In a relationship/living with partner	68	33	35
In a relationship/not living with partner	103	48	55
Other	1	0	1
Single	4	1	3
Household income			
£16 000–£29 999 a year	66	34	32
£30 000–£59 999 a year	95	44	51
Less than £16 000 a year	77	42	35
More than £60 000 a year	84	40	44
Prefer not to say	27	12	15
OCC engagement frequency			
Between once a month and once a year	120	59	61
More than once a month (but less than once a week)	87	44	43
Once a week or more	88	40	48
Rarely/never (no more than once a year)	54	29	25
Current mental health			
No	158	82	76
Yes	191	90	101
Current health			
No	300	152	148
Yes	49	20	29
COVID-19 diagnosis			
No	280	144	136
Suspected and recovered	30	8	22
Suspected and still ill	19	8	11
Yes diagnosed and recovered	14	9	5
Yes diagnosed and still ill	6	3	3
Isolation status			
I am leaving the house as normal	51	24	27
I am leaving the house for work/essential responsibilities	154	77	77
I am leaving the house only for essential supplies	95	44	51
I am not leaving the house at all	13	9	4
Prefer not to say	36	18	18
Antidepressant			
No	288	142	146
Yes	61	30	31
Baseline mental health			
No clinically significant symptoms	36	15	21
Mild	80	38	42
Moderate	105	54	51
Severe	128	65	63
Mental health at 6 weeks			
No clinically significant symptoms	137	70	67
Mild	76	38	38
Moderate	54	24	30
Severe	82	40	42

OCC, Online Cultural Content; WoB, Ways of Being.

Of the 349 participants who completed the exit questionnaire only 33 (less than 10%) were aged 16 and 17. Therefore, we lacked sufficient data to reliably compare those aged 16 and 17 with those aged 18–24. Of the 336 trial completers who reported location, only eight were located overseas. Therefore, we were unable to compare those based in the UK and those based overseas.

#### Data

Two online focus group discussions took place via Zoom, each lasting 60–90 min. The first focus group was attended by seven Ash participants and the second by six WoB participants. Further demographic information is provided in table 2.

**Table 2:** Characteristics of focus group participants

	Ash	WoB
Gender		
Male	1	2
Female	6	3
Other	0	1
Age		
16–17	0	1
18–24	7	5
Ethnicity		
White (British Irish or Other)	6	4
Asian British (Indian Pakistani or Bangladeshi)	1	0
Mixed race (Other)	0	1
Black/Black British (Caribbean African or Other)	0	1
Occupation		
Employment (not studying)	1	1
Studying and not working (including school)	4	3
Studying and working	0	1
Other	2	1
Relationship		
Single	3	2
In a relationship	4	4
Previous user of online culture		
Less than once a month	4	5
More than once a month	3	1
Mental health problem		
Current or past diagnosed depression or anxiety	3	3

WoB, Ways of Being.

Viewpoints were left anonymously and therefore we do not hold participant details for these. A total of 108 separate viewpoints were written in WoB. The majority were comments which stood alone; only a small number of participants responded to viewpoints from other participants. The majority of viewpoints were for the stories of Being Gwen, Being Tomoe, Being Ethel, Being Nur and Being Vernon.

Themes (and subthemes) identified in the qualitative analyses are shown in online supplemental table 3 with a narrative summary below.

#### Human connection

Participants felt a sense of human connection through their online cultural experience and described mental health and well-being benefits relating to this. This was described mostly by participants allocated to WoB. Participants allocated to Ash described some sense of connection, but this was mainly via art and artefacts. In WoB, the sense of connection was described both in relation to the protagonist (the artist or a person who had a close relationship with the art or artefact) via their stories, or with other participants, through reading their viewpoints. The connection many users felt to the protagonist was so strong that it transcended time and place, for example, *“The connection I felt with this woman from 2000 years ago whilst listening to her story is incredible. I had never heard of her before and I think that it is remarkable that such a powerful story is not told more often in the Western world.”* One of the participants who commented on Gwen’s story wrote: *“Gwen’s feelings of never being enough compared to a man resonate so strongly.”* Participants described that the viewpoints not only helped them feel connected to the content but also with other people engaging with the content which further reduced their feelings of isolation. Additionally, seeing others’ interpretation of stories provided context, made the mental health link in the stories clearer and exposed them to a greater range of perspectives, which was identified as being helpful to their own mental health.

Participants allocated to WoB in particular described the diversity of people in the stories as being positive as they found it reassuring that a range of people of different identities, backgrounds and cultures were recognised equally in the web experience. The inclusion of different types of people with different life experiences was suggested as providing the mental health and well-being benefits through a sense of comfort in acknowledging the existence of different types of people across the globe through history. In particular, the non-judgemental way in which the stories were told was seen as being helpful. For example, one WoB Focus group participant said, *“I think it was especially nice to see the diversity of people and experiences in all collections together. ‘Cause there’s definitely such a narrative when it comes to art and artists that there’s certainly an established canon of artists. And a lot of the people who were in the study would not necessarily be in that typically. It was nice and refreshing to see so many different kinds of people, all presented together equally. And a lot of people I hadn’t heard of, I was really surprised I hadn’t ‘cause I’d studied those periods so it was very interesting.”*

Seeing the stories and work of people of different backgrounds within WoB also provided inspiration to achieve or create things despite feeling different, or while experiencing challenges, for example, in the WoB focus group, one participant said *it’s just the acknowledgement that different types of people do exist and that if they can be comfortable with themselves and can still achieve all these things, then I should try and be more like that, in a way.* Participants even queried why stories like those in WoB are not told more frequently, for example, one participant wrote as a viewpoint about Vernon Lee, *She is like the renaissance artists, a polymath, and a huge inspiration for the queer community. Again, the question stands, why are people like this not taught in the main curriculum.*

Participants allocated to both interventions described that engagement with content about people of different backgrounds, characteristics or experiences that they could identify with had benefits through feeling acknowledged and reflected. For example, one WoB participant wrote this viewpoint *It’s interesting to see how LGBTQ relationships worked in the past—it’s nice to see similarities to my own relationship now. It shows that LGBTQ people, especially under-represented lesbians, have always been here.*

Feeling reflected was not only in terms of identities, such as ethnicity or gender identity, but also in terms of shared human experiences such as feeling different or marginalised. Challenging experiences continued to resonate particularly in hearing the stories about people who were marginalised. For example, a WoB participant wrote the following viewpoint, “*Today again the stories focused on the marginalised and outsiders in society, especially the displaced and their ability to embrace their own identities in these new environments. This provided a promising and inspiring outlook onto my own today. Putting these ideas and experiences into perspective has been both humbling and inspired me to try to apply the same thinking to my own day today.”*

Participants described that non-judgmental stories told in a clear but warm way about different people and the ability to see other people’s viewpoints facilitated the feeling of a shared experience that allowed them to look outwards. They described engaging with WoB as being similar to therapy ‘*only better’* as there was a more outward focus, which they described as being more beneficial for mental health. They described that understanding and accepting themselves through a natural journey of discovery—without forcing them to focus on mental health issues that made them feel more like there is ‘*something wrong’* with them—was a positive of WoB. They described that, in comparison to therapy specifically focusing on their mental health issues, which could be confronting, self-acceptance through WoB occurred more organically and *on (my) own terms*.

Connecting with others across time and space via human stories, and with other participants, by reading their viewpoints was described as bringing perspective, which in turn were perceived to bring about positive emotional impacts. For example, one WoB participant wrote the following viewpoint, *“Today brought even more new perspectives and joys. I particularly enjoyed the many stories about the lives and times of women, and how they carved their own paths in different, but equally strong and inspiring, ways.”* Some participants reported that reflecting on other people’s experiences had benefits including feeling inspired, for example, as described in this WoB viewpoint, *“All of these stories were very inspiring. A lot of people here did not conform to societal expectations, whether it be through gender, sexuality, race or general social norms. None of them let their differences hold them back from pursuing what they wanted, showing that it is not impossible to achieve what you want if you set your mind to it and don't sway from what you believe in.”*

Participants identified that the human connection achieved via human stories as the key element for engagement and immersion providing a welcome distraction from the stress of life. This was described in the WoB focus group, *“… you appreciate the work more because you know the back story and what caused it. And it stopped just being an object. It kind of had a story and you got more connected to it. As opposed to in a museum, there’s lots and lots of exhibits and it’s a bit impersonal, and you kind of glaze over a bit and stop appreciating the art as much. And you kind of forget there was a person with a life and issues behind every single thing in there*.” As another WoB focus group participant said, “*I do think I wouldn’t have been as immersed if it weren’t stories about people. I think for me that was a big part of the attraction”*.

In Ash, participants observed that the artefacts were the ‘hook’ that drew them in and motivated them to learn about things they would not otherwise have been drawn to. This lacked the deep, varied and frequent mental health benefits described by WoB participants via human connection.

#### Content/journey

Learning new information was more frequently referenced as a positive aspect of the intervention by WoB participants than by Ash participants. For example, one WoB participant wrote, *“I enjoyed learning about artists and works that I hadn't come across before. I love art, and literature, and exploring the resource was interesting.”* WoB was highlighted by participants for its social connection element that augmented the learning experience. Learning was described as an enjoyable adventure, one WoB participant writing the following viewpoint, *“These stories really lifted my mood and inspired me today, making me feel as though I was actually visiting the countries and learning about those different cultures at a time when that is not possible. Thank you for it!”* WoB users described learning about artists that they had not heard before or aspects of their personal stories they were unaware of. Several commented on how enjoyable it was to learn new things and how inspiring it was to discover more about these artists, artistic movements and historical eras, one saying “*I enjoyed my time engaging with my resource and learnt new things.”*

For those allocated to WoB, there was a general sense of excitement about finding new information and discovering new stories. WoB participants reported a feeling of achievement and satisfaction attained through acquiring new knowledge through the intervention. Ash participants also described enjoyment in exploring new content “*I was happy with the Ashmolean site as not been to the Ashmolean museum before and enjoyed exploring.”* Interestingly having a familiar touchpoint also inspired interest in both interventions, for example, an Ash Focus group participant said, “*I felt like a point of familiarity was a really good way to into learning about something that I would not have initially been drawn to.”*

#### Features

In WoB, participants noted that the web experience was well made and this, in and of itself, was described as bringing about mental health benefits. WoB was compared with many mental health resources such as leaflets, which were felt to be of poor quality. WoB was also described as being a welcome change—to have something that felt that it had been tailored to the users’ experience, thought about and made to a high-quality standard. The structure of WoB and its curation in general were described as inducing feelings that participants were cared for and supported and made the experience feel more immersive. Participants also commented that quality of WoB led to mental health benefits via other mechanisms, for example, a WoB focus group participant said, “*It was so well-made, it was very motivating to use.”*

Participants allocated to both interventions said that the long form content was out of keeping with much of their usual screen time, but that it was beneficial to their mental health as they found it more absorbing, engaging and immersive. For example, one Ash focus group participant said “(usually it) *felt not using my brain power either just chatting with friends or games. This engaged my brain power more. It was different to my normal screen time.”*

The optionality of different modalities to interact with the stories (look, read, hear) were welcomed by participants. They liked that the stories could be absorbed in different ways according to their state of mind and preference at the time. Many commented on the benefits of the audio feature of a consistent ‘*real*’ voice narrating the human story which they found *calming*, it further promoted inclusivity and helped them absorb the information better. Additionally, they found the colours immersive and soothing.

#### Setting/when used

The interventions and guidance around usage were described as providing a sense of structure to their day for participants allocated to either intervention. This was experienced as beneficial at a time when participants had to spend a lot of time at home due to the COVID-19 restrictions that increased during that time. Participants described that their interventions gave them ‘*a small sense of purpose’* and something to look forward to. Engaging with their intervention was described as different to their normal screen time. It was described as a downtime activity that was still productive. One Ash participant described it as *“still engaging in something I was interested in but was more productive than scrolling through Facebook.”*

Many participants, allocated to either intervention, viewed flexibility of when and where they could use their intervention (because they were available online) as positive. For example, one WoB participant said *“Online gives you the flexibility. I can be doing it from bed. I can be doing it with a cup of tea and I think that really…if you’re doing it for a distraction or soothing, then that’s key.”*

Participants described that they used their intervention between activities, as a downtime activity, or to allow their mind to recover from one activity before moving to another, for example, one WoB participant said that it “*Helped me like a buffer to stop and look at things and engaged with the website more in a structured way which was helpful.”* Some used their online arts and culture intervention explicitly to relax, one WoB participant commenting that “*It would help me relax, learn more, and feel more connected to other people.”* Many participants described benefits of using it at the end of the day and before bed and some said that it aided getting to sleep or sleep quality.

#### Mental health impact- Positive

Participants allocated to WoB described wide-ranging mental health benefits. Increased motivation was recognised as a benefit that led to increased social interaction and creativity. For example, one said that WoB offered food for thought about people who led to engaging conversations with their housemates. Another shared that WoB *“Greatly improved my ability to deal with things and communicate with my peers more closely.”* This appeared to relate to increased human connection and was more commonly described in those allocated to WoB compared with Ash.

Both interventions were perceived as an active rather than passive way to spend time. Participants noted that the interventions deterred them from spending time on social media evoking feelings of achievement derived from learning something new, unrelated to their work or studies, which was viewed positively. This in turn was described as positively impacting participants in terms of fostering inspiration and motivation to pursue interests or establish new routines. WoB and Ash were both identified as encouraging creativity through inspiration and motivation. One WoB participant noted that *it was noticeable that I felt more inspired to go and do things and more motivated for a few hours after engaging with the content*. Another mentioned that they were inspired to pursue an art history degree. Ash participants also identified feelings of creativity evoked through using the intervention and remarked on how it motivated them to work on other aspects of their lives such as schoolwork. One participant noted that the intervention inspired them to get out of their ‘inner world’ and helped them feel that the world is a better place.

Moreover, these interventions were described as providing engaging cultural experiences, with WoB being described as immersive, effectively diverting participants’ focus from daily concerns (eg, work-related or study-related issues) to a calmer state of mind. Positive impacts on sleep were also reported. Both interventions were described as a distraction from everyday life, shifting participants’ focus away from their usual activities such as studying or work to engage in calming activities which facilitated improved sleep.

#### Neutral or negative effects

Structure and navigation were described as a drawback for Ash participants while these features were generally commended by WoB participants. Lack of structure and ‘*too much choice’* was described by many participants as reducing potential mental health benefits. One participant noted however that they were able to overcome this and eventually enjoy the exploration: *“I felt spoilt for choice in the beginning, perhaps would have liked to have more directions in the first instance. But once started to explore you find it’s like going down a rabbit hole and you find what works for you.”* However, in many cases, participants described that too much content in too many different formats with insufficient guidance overall had a neutral or negative mental health impact. Many Ash participants described feeling overwhelmed, for example, one participant described that *‘There were too many choices, which is usually good, but I just lost some time trying to decide what to click on. I think I actually felt a bit of FOMO with regard to the content I didn't click on.’*

Some participants described that they would have appreciated more of an opportunity to have their own voice heard, with “*input and chances to elaborate feelings”*. Some also wondered about the relevance and benefit of much of the content to themselves and their mental health and would have appreciated more of a bespoke experience, guidance or explanations of how to gain benefit. In contrast to WoB, Ash was not identified as immersive. This could be attributed to challenges participants described in navigating the Ash website. One Ash participant reported that *“Sometimes navigating the webpages was difficult, which detracted from the relaxing effects it can have.”*

## Discussion

The main themes identified as relating to the mental health impact of online arts and culture by YP were of human connection, the content and journey of the online experience, the features, setting and when it was used. Positive mental health impacts as well as neutral/negative effects were identified. Positive mental health impacts were often described in association with human connection and were more commonly described by participants allocated to WoB, subthemes included socialisation, motivation and focus. Neutral and negative effects were more commonly described by participants allocated to Ash and included subthemes of being overwhelmed and technical problems.

Human connection was seen as essential in terms of how engaging, absorbing and immersive the experience was and pivotal to the mechanisms relating to mental health benefit. This was more commonly reported in WoB participants and is in keeping with the quantitative analysis, which demonstrated that WoB was more effective in reducing negative affect than Ash. It is also notable that diversity and inclusion were seen as essential within the human connection element. A range of stories about different people (some of whom are like them) appeared important in allowing people to see that it was ‘OK to be different’, and that there are a range of identities, which are all OK. This appeared to be noted as important across participants of WoB and also for some Ash participants who had explored diverse human stories as a part of their experience. This is consistent with the literature describing the negative impact of exclusion from culture by gender, class and race in cultural occupations, which ultimately leads to reduced diversity in collections.^34^

Being able to identify with the people in stories was mentioned by many people who also talked about reflecting on their own identity, particularly gender identity and sexuality. The benefits of feeling reflected may also apply to people of different ethnic backgrounds and neurodiversity. This is in keeping with results of the quantitative analysis, which identified that WoB was particularly efficacious in people of different ethnic backgrounds. A sense of connection,^35^ community,^36^ belonging and identity^37^ is thought to be protective to mental health in people of under-represented identities and to promote resilience. This may be particularly important in YP who are feeling increasingly disconnected from their communities.^38^ This also indicates that WoB may function as an indicated prevention strategy and may be particularly effective in people of under-represented identities.

Many YP allocated to WoB liked being able to look outwards rather than inwards and form their own perspectives due to the non-judgemental and neutral tone of the stories. They commented on the non-threatening nature of self-discovery and particular noticed that it was preferable to therapy in which they feel forced to look inwards and notice problems to inspire change. This may indicate that there was a benefit from being given the opportunity to think about the internal experiences of the people in the stories and how this impacted on their decision-making and behaviour. In psychological terms, this may indicate that the opportunity to mentalise was beneficial and is consistent with many studies in which mentalisation is conceptualised as a mediator of psychopathology.^39^

The benefits of a ‘shared experience’ in being able to write their own and read other people’s perspectives in the ‘viewpoints’ feature was also experienced as being helpful to mental health by allowing them to recognise that other perspectives can exist and in introducing different avenues for mental health benefit. In previous studies, YP have described the evolution of depression in adolescence as noticing changes in their relationship with others and changes in their relationship with the world.^40^ It is possible that the aetiology of depression in adolescence is different to adulthood with the need for a sense of identity and self-actualisation competing with the pressure to conform to social and societal expectations. This may explain why conventional forms of therapy, such as CBT that mainly rely on analysis and self-improvement, developed in working age adults, are not as acceptable or effective in adolescents.^41^ While attempts are made to adapt therapies to people of specific backgrounds,^42^ many YP described benefits of identifying with, understanding and deriving therapeutic benefit from relating to other humans who had also experienced marginalisation, the feeling of not fitting in or of having to pave a way for themselves in difficult circumstances in different times and settings. This suggests that a process of self-realisation and self-identification may be of importance and the sense that other people have also had similar experiences may reduce feelings of isolation.

### Implications

The strategy of using online arts and culture as an early intervention or prevention strategy for mental health problems in under-represented YP is promising. Use of mixed methods and participatory designs to identify impact on mental health and potential mechanisms. The identification of potential mechanisms could be used to optimise and target these approaches further.

### Limitations

The study took place in 2020/2021, which was a period that had specific challenges for YP^43^,^45^ and particularly those from under-represented backgrounds.^45^ It is possible that online sources of support were more acceptable during that period, however increased use of technology has been viewed both positively and negatively. Since COVID, it has been recognised that online support appears promising but requires further robust evaluation.^46^

## Supplementary material

10.1136/bmjopen-2025-105217 online supplemental file 1

10.1136/bmjopen-2025-105217 online supplemental file 2

10.1136/bmjopen-2025-105217 online supplemental file 3

## Data Availability

No data are available.
