# Supplementary material for: Online cultural experiences for mental health in people aged 16–24: a qualitative analysis of multisource data from a randomised controlled trial
Source: BMJ Open. 2026 Apr 28;16(4):e105217. doi: 10.1136/bmjopen-2025-105217 (PMC13140964; doi:10.1136/bmjopen-2025-105217)
Supplement: online supplemental file 1 [file bmjopen-16-4-s001.docx]

**Area of Inquiry**

How often did you use it? Intense phase? After the intense phase?

Was it helpful for MH?

Was it detrimental?

Were there any other reasons for using/not using it

Any particular bits

In what way?

Was there enough there to look at?

Was the recommended time to spend on it in first phase optimal (should be more/less)

Should we have guided time to spend on it

How would you recommend it’s use/testing into the future

**WoB-**

Did having a passcode effect your experience of the intervention (did you use it more or less because of this)

How did you experience the viewpoints on the experience?

What do you think about being given the opportunity to give a viewpoint?

How could this be optimised for mental health/wellbeing?
