# Supplementary material for: Online cultural experiences for mental health in people aged 16–24: a qualitative analysis of multisource data from a randomised controlled trial
Source: BMJ Open. 2026 Apr 28;16(4):e105217. doi: 10.1136/bmjopen-2025-105217 (PMC13140964; doi:10.1136/bmjopen-2025-105217)
Supplement: online supplemental file 3 [file bmjopen-16-4-s003.docx]

**TABLE 3: Themes and subthemes**

| **Theme** | **Subtheme** | **Example Quote** | **Arm** | **Source** | **Gender, ethnicity, occupation** | **Study ID** |
| --- | --- | --- | --- | --- | --- | --- |
| Human connection | Diversity/range/inclusivity | The stories here are really inspiring - the range of backgrounds, identities, times, and places all combine to give an impression of a wide world which everyone can play a part in | WoB | Viewpoint | N/A | N/A |
|  | Seen/represented/reflected | I was interested in the LGT artefacts and would not have ever been drawn to what was in the list but the way into that was the LGT connection and is really important as sometimes it feels like an absence of historic LGT so felt connected, seen and heard also acknowledged in this segment. | Ash | Focus group | Male, 18-24, White, Employed | 952438 |
|  | Perspective/ reflecting/ looking outwards | Again, I think it’s really valuable being able to see other people interpret things. It kind of gets you out of your own head and experiences and gives you perspectives you wouldn’t normally have otherwise. I think this is quite important especially in terms of connecting with other people – ‘cause sometimes it can be hard. You don’t know what they’ve gone through. Being able to read it in their own words, yeah, it… | WoB | Focus group | Male, 18-24, White, Studying and working | 791056 |
|  | Empowering/ inspiring | It's very inspiring to read about women who challenged beauty standards - it shows that everything is just dependent on the era! | WoB | Viewpoint | N/A | N/A |
|  | Engaging/immersive/distraction | It was a good distraction from the typical stress of my day but in a way that made me still feel like i had achieved/ learned something rather than just wasting time. | WoB | Free text | Female, 18-24, White, Studying and not working | 521689 |
|  | With art/artefacts | Very easy to feel disconnected and isolated at home and thought this was really important and a transformative experience by being able to connect with things and objects that are outside of our very familiar environments at the moment | Ash | Focus group | Male, 18-24, White, Employed | 952438 |
| Content/journey | Learning/exploration/discovery | I enjoyed learning about artists and works that I hadn't come across before. I love art, and literature, and exploring the resource was interesting. | WoB | Free text | Female, 18-24, Mixed Race, Studying and not working | 327415 |
|  | Novelty | Nice pleasant change from the kind of other regular things that I was doing. It was something to look forward to. | Ash | Focus group | Female, 18-24, Asian British, Studying and not working | 366654 |
|  | Familiarity | It was my connection in terms of the familiarity with the objects that I had studied.  It was a Greek embroidery which reminded me of my grandmother who had these objects and embroidery decorated in her house. This is how I felt the connection. | Ash | Focus group | Female, 18-24, White, Studying and not working | 368941 |
| Features | Well made | I think because it was so well made and you could tell people put care into it, it made you want to interact more and appreciate it more. Just in my experience, comparing it to mental health leaflets that are not normally that well-made, they’ve been thrown together and people don’t care, you can feel a bit ‘oh they don’t care’ but in this case, ‘cause it was so well-made  you felt kind of supported in a way. | WoB | Focus group | Female, 18-24, White, Studying and not working | 327415 |
|  | Long-form | Enjoyed the long form text and the image description parts more, reading about the history books and origins | Ash | Focus group | Male, 18-24, White, Employed | 952438 |
|  | Multi mode/audio | I really appreciate that the website gave the option to listen to all the articles in audio format. That made me feel more connected to the person the story was about, even though it wasn't them narrating it. | WoB | Free text | Female, 18-24, White, Employed | 922454 |
| Setting/ When used | Structure | the intervention helped me very much as having something fixed on a morning and evening schedule and being able to coordinate even just regular chores around something that is fixed on my day schedule, gave a me small sense of purpose. | Ash | Focus group | Female, 18-24, White, Unemployed | 983584 |
|  | Flexibility/accessibility | it’s nice to be able to pick into other people’s collections and learn about them without having to travel there and to be able to do that in the middle of the night in your bed is just, I think, beneficial | WoB | Focus group | Female, 18-24, White, Studying and not working | 521689 |
|  | Downtime | It was something I did at the end of the day and it took my mind off anything that happened during the day and I felt like I had done something to…a form of self-care. I’d learnt something and I’d actually taken away something from it. | WoB | Focus group | Male, 18-24, White, Studying and working | 791056 |
| Mental health impact | Socialisation | I have become more frequent in contact with others, waiting to get along with friends around me.The quality of sleep becomes higher. | WoB | Free text | Male, 18-24, White, At school | 278170 |
|  | Motivation/proactivity/creativity | Greatly improved my ability to deal with things, and communicate with my peers more closely. | WoB | Free text | Male, 18-24, White, At school | 631078 |
|  | Focus/ calm/ mood | it held my focus in a way a lot of things could not. It kept me interested, and learning something new gave me a sense of achievement and satisfaction. | WoB | Free text | Female, 18-24, White, Studying and not working | 327415 |
| Neutral/negative effects | Overwhelmed/uncertain | Perhaps a "feature of the day" or "chosen for you" type function? There are a lot of choices to pick from on the Ashmolean from home section of the website, so spotlighting one particular story/video/jigsaw at the top of a page may provide a useful first step for engagement, and make it easier to decide. Mental health and choice paralysis seem to come hand in hand sometimes. | Ash | Free text | Female, 18-24, White, Studying and not working | 632856 |
|  | Irrelevance | some of the things on the website were not necessarily things that were stimulating for our age group, so may not connect with it in a way that will help with mental health | Ash | Focus group | Female, 18-24, White, Studying and not working | 595929 |
|  | Technical problems | The website itself was very glitchy which sometimes stressed me out and increased my anxiety as I tried to give feedback but the website wouldn't allow it. | WoB | Free text | Female, 18-24, White, Studying and not working | 873014 |
| **Legend,**  **Free text: Exit Survey free test**  **Viewpoint: Ways of Being viewpoint:** | | | | | | |
