# Supplementary material for: Online cultural experiences for mental health in people aged 16–24: a qualitative analysis of multisource data from a randomised controlled trial
Source: BMJ Open. 2026 Apr 28;16(4):e105217. doi: 10.1136/bmjopen-2025-105217 (PMC13140964; doi:10.1136/bmjopen-2025-105217)
Supplement: Supplementary file 4 [file bmjopen-2025-105217.reviewer_comments.pdf]

PEER REVIEW HISTORY

BMJ Open publishes all reviews undertaken for accepted manuscripts. Reviewers are asked to complete a checklist review form and are provided with free text boxes to elaborate on their assessment. These free text comments are reproduced below.

ARTICLE DETAILS

Title (Provisional)

Online cultural experiences for mental health in people aged 16-24: A qualitative analysis of multisource data from a randomised controlled trial

Authors

Sheriff, Rebecca; Riga, Evgenia; O'Dell, Bessie; Chandler, Louise; Adams, Helen; Glogowska, Margaret

VERSION 1 - REVIEW

**Reviewer**	**1**
**Name**	**Ibrahim , Nashwa**
**Affiliation**	**Mansoura University**
**Date**	**25-Nov-2025**
**COI**	**None**

This study is very interesting, and I have enjoyed reading it, I got some suggestions for authors to address:

Abstract

The first 4 sentences in the abstract are more of methods, please add two background sentences

Introduction

Lines 10-11; Three quarters of all mental disorders emerge before the age of 24 (Kessler et al., 2005). Authors relied on data from a study which dated back to more than 20 years ago, pls use more recent stats

I would suggest that authors add to the background section few sentences on the significance and value of different online cultural resources on mental health

Sampling

Please provide a rationale on: Sampling Trial participants were aged 16-24 and based in the UK (or 18-24 if based overseas

In the results

Pls add more participants' quotes in each theme

Pls describe themes in terms of strengths (which one got more participants' quotes)

Is there differences between participants who are based in the Uk and those who are based overseas in the results?

**Reviewer**	**2**
**Name**	**Mayer, Gwendolyn**
**Affiliation**	**University Hospital Heidelberg, Internal Medicine and Psychosomatics**
**Date**	**02-Dec-2025**
**COI**	**None**

This paper presents a qualitative study done within a proof-of-principle RCT on two different kinds of online cultural interventions for mental health prevention in young people. The study is innovative and interesting and the paper is well-written. Few details will help to improve it:

1. Although it is clear that the thematic analysis was also used to explain the quantitative results of the RCT, there is no detailed explanation of how this was done and to which of the measures collected in the RCT they refer exactly. These results are mentioned rather arbitrarily in the discussion. However, the analytical approach should be described in such a way that it is fundamentally repeatable.

2. Did you detect any age differences in the results?

3. The relevance of mental health prevention for adolescents is undoubtedly high. However, apart from participating in a study, the online culture presented on museum websites may not be the first choice for young people. Suggestions or even findings from the literature on the implementation of such measures would be helpful.

4. p. 6, line 15: correct the wrong quotation format: "RCT [40]"

5. limitations are missing so far (though mentioned in the SRQR -list)

VERSION 1 - AUTHOR RESPONSE

Reviewer: 1
Dr. Nashwa Ibrahim , Mansoura University
Comments to the Author:
This study is very interesting, and I have enjoyed reading it, I got some suggestions for authors to address:
Thank you

Abstract

The first 4 sentences in the abstract are more of methods, please add two background sentences
Thank you. In the journals Author guidance, the format suggested for the abstract is Objective, Methods, Results, Conclusions. We have therefore moved the noted sentences to methods as suggested (but have not added background sentences, as background does not form a part of the abstract in the BMJ)

Introduction
Lines 10-11; Three quarters of all mental disorders emerge before the age of 24 (Kessler et al., 2005). Authors relied on data from a study which dated back to more than 20 years ago, pls use more recent stats
Thank you. We agree. We have added further references as per this helpful suggestion

I would suggest that authors add to the background section a few sentences on the significance and value of different online cultural resources on mental health
Thank you. We agree. We have added a further paragraph as per this suggestion
'Evidence suggests that culture and the arts impact positively on mental and physical health and wellbeing. (Fancourt et al., 2019, Fancourt et al., 2021, Fancourt and Finn, 2019, Van Lith et al., 2013, Camic and Chatterjee, 2013, Callinan and Coyne, 2020) The mechanisms by which this might occur include emotional activation, aesthetic engagement, interaction, social and cognitive stimulation, sensory activation and imagination (Fancourt et al., 2019, Callinan and Coyne, 2020).However, engagement with culture and the arts appears to be poorest for those who might benefit the most. (Syed Sheriff et al., 2022a)'

Sampling
Please provide a rationale on: Sampling Trial participants were aged 16-24 and based in the UK (or 18-24 if based overseas
Thank you, we agree. The following sentence has been added
'In the UK, young people aged 16 and 17 years are able to give informed consent without parental involvement as 'competent youths'.

In the results
Pls add more participants' quotes in each theme
We have added participant quotes per subtheme in the tables and further quotes in the text to exemplify and illustrate the points we are making within the text to give a rich expression of the range and expression of viewpoints.

Pls describe themes in terms of strengths (which one got more participants' quotes)
Our qualitative research MG has responded to this query

Quotations from participants are included to illustrate and enrich the themes which emerge from the data and to contribute to the transparency of interpretations of the data made by the researchers. In qualitative research, this is not a numerical consideration. We do not include quotations to represent the 'strength' of the themes displayed or the numbers of participants sharing that view. We would include a quotation to demonstrate the expression of the view (usually from one participant), as it is range and diversity of views and perceptions we are seeking rather than the number of times the view was expressed.

Is there differences between participants who are based in the Uk and those who are based overseas in the results?

We did not have sufficient data to compare those based in the UK and those based overseas. We have added in the following text
'Of the 336 trial completers who reported location, only eight were located overseas. Therefore, we were unable to compare those based in the UK and those based overseas.'

Reviewer: 2
Dr. Gwendolyn Mayer, University Hospital Heidelberg
Comments to the Author:
This paper presents a qualitative study done within a proof-of-principle RCT on two different kinds of online cultural interventions for mental health prevention in young people. The study is innovative and interesting and the paper is well-written. Few details will help to improve it:

1. Although it is clear that the thematic analysis was also used to explain the quantitative results of the RCT, there is no detailed explanation of how this was done and to which of the measures collected in the RCT they refer exactly. These results are mentioned rather arbitrarily in the discussion. However, the analytical approach should be described in such a way that it is fundamentally repeatable.
Thank you for these comments. More has been added to describe the data collection timepoints compared to the quantitative data collection and in addition more detail regarding the analytical approach has been added.

Data collection
Quantitative data was collected at baseline, daily on three consecutive days, at day 5 and week at 6. The qualitative data described in this study comprises three sources of data, viewpoints, free text responses and focus groups.
'Viewpoints were a feature of WoB that allowed participants to write text anonymously reflecting on content which was then made available alongside other viewpoints for other participants to read.

Free-text responses were collected at the six-week follow-up exit assessment of the trial. All participants had free-text options to leave feedback on their allocated online cultural experience at the six-week timepoint (Exit). There were two sets of free-text response questions. The first set was relevant to the intervention. Participants were invited to share any noticeable positive or negative impacts of their allocated cultural experience and their suggestions on how their allocated intervention could be improved for mental health. The second set of questions sought participant views on their research experience and any suggested improvements. Feedback on the research itself will not be reported here.'

'Deductive thematic analysis was utilised to explore possible links to the quantitative findings of the RCT (Syed Sheriff et al., 2022b), in particular negative affect scores across the intervention phase and at six weeks, as well as previous work on potential mechanisms (Syed Sheriff et al., 2023)'

2. Did you detect any age differences in the results?
Thank you for this comment. Unfortunately, we did not have sufficient data to meaningfully compare participants based on age. We have therefore added in the following text
'Of the 349 participants who completed the exit questionnaire only 33 (less than 10%) were aged 16 and 17. Therefore, we lacked sufficient data to reliably compare those aged 16 and 17 with those aged 18-24.'

3. The relevance of mental health prevention for adolescents is undoubtedly high. However, apart from participating in a study, the online culture presented on museum websites may not be the first choice for young people. Suggestions or even findings from the literature on the implementation of such measures would be helpful.
Thank you for this comment. We agree. We have added in the following text
'However, engagement with culture and the arts is poorest for those who might benefit the most.21 During consultations in our work, people with lived experience of mental illness said that this was not due to lack of interest. They thought that those with lived experience should be an active part of any strategy to optimise such resources for mental health benefit in young people'.

4. p. 6, line 15: correct the wrong quotation format: "RCT [40]"
We agree. This has been completed

5. limitations are missing so far (though mentioned in the SRQR -list)
Thank you. The following has been added
'The study took place in 2020/2021 which was a period that had specific challenges for YP (Montero-Marin et al., 2023, Crosby L et al., 2020, Eboigbe et al., 2023) and particularly those from underrepresented backgrounds (Eboigbe et al., 2023). It is possible that online sources of support were more acceptable during that period, however increased use of technology has been viewed both positively and negatively. Since COVID it has been recognised that online support appears promising but requires further obust evaluation. (Fischer-Grote et al., 2024)'

VERSION 2 - REVIEW

**Reviewer**	**2**
**Name**	**Mayer, Gwendolyn**
**Affiliation**	**University Hospital Heidelberg, Internal Medicine and Psychosomatics**
**Date**	**08-Feb-2026**
**COI**	

Thank you very much, I have no further comments.
